# The Relevance of Follicle Number and Androgens in Metabolic Alterations in Polycystic Ovary Syndrome: A Cross-Sectional Study

**DOI:** 10.3390/jpm16100512

**Published:** 2026-09-30

**Authors:** Nicoletta Cera, Joana Pinto, Marica Tina Maccarone, Duarte Pignatelli

**Affiliations:** 1 Faculty of Psychology and Education Sciences, University of Porto, 4200-135 Porto, Portugal; pinto.joanafd@gmail.com; 2 Research Unit in Medical Imaging and Radiotherapy, Cross I&D Lisbon Research Centre, Escola Superior de Saúde da Cruz Vermelha Portuguesa, 1300-125 Lisbon, Portugal; 3 Faculty of Medicine, University of Porto, 4200-319 Porto, Portugal; dpignatelli@yahoo.com; 4 AUSL Pescara, “Santo Spirito” Hospital of Pescara, 65124 Pescara, Italy; maccaronemarica@hotmail.com; 5 Department of Endocrinology, Centro Hospitalar Universitário de São João, 4200-319 Porto, Portugal

**Keywords:** PCOS, follicle number, PCOM, phenotypes, insulin resistance

## Abstract

**Background/Objectives:** Polycystic Ovary Syndrome (PCOS) is characterized by reproductive and metabolic disturbances, including insulin resistance and altered ovarian morphology. The clinical manifestations of PCOS include metabolic, reproductive, and psychological issues that negatively affect quality of life, making PCOS a significant public health concern. The relationship between follicle number and metabolic alterations across PCOS phenotypes still remains unclear. **Methods:** We conducted a retrospective cross-sectional study of 289 PCOS patients and 50 controls, assessing insulin resistance, lipid profiles, ovarian volume, and follicle count across phenotypes A, B, and C. We compared the three PCOS phenotypes and identified differences in follicle number, ovarian volume, insulin-related biomarkers, androgens, and LH and FSH. **Results:** Phenotype A exhibited higher insulin resistance and adverse lipid profiles compared to controls. The follicle number significantly discriminated between phenotypes and is associated with insulin-related alterations, one of the most clinically relevant aspects of PCOS. It is also correlated negatively with insulin resistance, most notably in phenotype B. Ovarian volume even differed among phenotypes but showed weaker metabolic associations. There is also a significant relevance of ovarian dysmorphology in PCOS and its association with hyperinsulinemia and insulin resistance. **Conclusions:** There is a complex interplay among the ovarian morphology, androgen and metabolic patterns in the different PCOS phenotypes. Follicle number is a relevant marker linked to insulin resistance in PCOS, particularly in phenotype B, supporting its role in clinical phenotyping and potential for guiding personalized management strategies.

## 1. Introduction

Polycystic Ovary Syndrome (PCOS) is a common pathological condition affecting up to 21% of women of reproductive age [1,2]. The diagnosis of PCOS is based on the presence of clinical and/or biochemical hyperandrogenism, oligo-anovulation, and polycystic ovarian morphology (PCOM). According to ultrasonographic criteria, the presence of at least 12 follicles with a maximum diameter of 2–9 mm or an ovarian volume greater than 10 mL is required [3]. Additionally, other causes of hyperandrogenism, such as nonclassical congenital adrenal hyperplasia, idiopathic hyperandrogenemia, and hyperprolactinemia, must be excluded [4].

Based on these criteria, PCOS can be categorized into four phenotypes [5,6]. Phenotype A includes patients with oligo-anovulation, clinical/biochemical hyperandrogenism, and PCOM. Phenotype B presents oligo-anovulation and clinical/biochemical hyperandrogenism, while phenotype C shows hyperandrogenism and PCOM. Phenotype D is characterized by oligo-anovulation and PCOM.

In addition to the existence of different diagnostic criteria, the etiology of PCOS remains unclear. Multiple factors, including genetics, environmental influences, and lifestyle choices, may contribute to the underlying alterations observed in PCOS, such as insulin resistance, androgen excess, and adipose tissue dysfunction [7].

The clinical manifestations of PCOS include metabolic, reproductive, and psychological issues that negatively affect quality of life, making PCOS a significant public health concern. PCOM is one of the principal characteristics of PCOS and has been associated with reproductive issues and hyperandrogenism [8,9]. Several studies have reported an association between PCOM and hyperinsulinemia [10,11,12], which plays an important role in blocking dominant follicle selection, resulting in the characteristic “string of pearls” ultrasound sign, with preantral follicles distributed throughout the ovarian parenchyma with a peripheral predominance [7,13].

Alterations in lipid metabolism are also observed in PCOS, including increased cholesterol and triglyceride levels and reduced high-density lipoprotein (HDL). These three serum biomarkers are still considered the gold standard for assessing lipid alterations in women with PCOS [14]. Blood lipids are essential for folliculogenesis in the ovaries. Cholesterol derived from lipoproteins (LDL and HDL) is crucial for hormone synthesis, including androgens that are converted into estrogen and progesterone. Theca cells absorb these lipoproteins to support follicular growth. Additionally, fatty acids in blood and follicular fluid provide energy for oocytes and granulosa cells. Elevated oleic acid levels can disrupt lipid profiles and induce inflammation, while high arachidonic acid levels may impair mitochondrial function and promote apoptosis in granulosa cells [15,16].

Granulosa cells are essential for oocyte growth and maturation, providing energy substrates due to the oocyte’s limited glycolytic capacity. Mitochondria in these cells are critical for ATP production, steroid hormone synthesis, and redox regulation. Glycolytic activity supports primordial follicle recruitment and growth through mTOR signaling, highlighting its role in folliculogenesis. In women with PCOS, particularly those who are obese, impaired glycolytic activity and mitochondrial dysfunction in granulosa cells limit energy production, compromising follicular maturation and oocyte quality [17]. Abnormal systemic lipid metabolism in PCOS can lead to lipotoxicity, oxidative stress, and inflammation, negatively affecting hormone production and resulting in stagnant microfollicles. Hyperinsulinemia due to insulin resistance also impacts follicle development, promoting androgen production and hindering maturation, which can lead to the formation of small cysts typical of PCOM [18,19,20].

The present study aims to assess the association between insulin resistance, lipid alterations, ovarian volume, and follicle count in a cohort of women affected by PCOS. In particular, we aim to investigate this association across PCOS phenotypes.

## 2. Materials and Methods

We conducted a retrospective cross-sectional study using data from women who attended endocrinology consultations at the University Hospital “São João” (CHUSJ) in Porto, a tertiary academic medical center, between 2010 and 2022. The study was approved by the local ethics committee (Protocol No. 23000471, approved on 10 January 2023). Patient consent was waived due to the retrospective nature of the study and the use of pseudo-anonymized clinical records, under the institutional protocol approved by the Responsible for Access to Information (RAI) and the Ethics Committee of Centro Hospitalar Universitário de São João (CHUSJ). 

### 2.1. Participants

All patients with Polycystic Ovary Syndrome (PCOS) who attended endocrinology consultations at CHUSJ between 2010 and 2022 were thoroughly evaluated using a standardized protocol. The same protocol was applied to control women who were voluntaries searching for a routine check-up without oligomenorrhea, hirsutism, or other characteristics of PCOS. In the selected control cases, the existence of anovulation or hyperandrogenism was excluded. The exclusion criteria for all participants were use of combined oral contraceptives or other hormonal treatments, menopause (defined as the absence of menstruation for at least 12 consecutive months after the age of 45), thyroid dysfunction, hyperprolactinemia, Cushing’s syndrome, and ovarian or adrenal tumors. The participants included in the control group, in addition to not having a diagnosis of PCOS, did not have any of the above-mentioned exclusion conditions. Consecutive patients and controls were recruited to minimize sampling bias.

PCOS was diagnosed when women met at least two of the following three criteria:

Clinical hirsutism or hyperandrogenemia: Elevated androgen levels, defined as total testosterone (T) ≥ 0.73 ng/mL, free testosterone (T) ≥ 2.5 pg/mL, androstenedione ≥ 3.1 ng/mL, or dehydroepiandrosterone sulfate (DHEAS) ≥ 4300 ng/mL. These levels were determined using a Chemiluminescence Immunoassay (CLIA).

Oligoanovulation: Fewer than 9 menstrual cycles per year. In women with regular cycles, oligoanovulation was confirmed by low progesterone levels during the expected luteal phase, assessed on at least two separate occasions.

Polycystic Ovarian Morphology (PCOM): Defined sonographically by at least one of the following criteria: ≥12 follicles in at least one ovary in sagittal/2D equatorial plane evaluation with a follicle diameter ranging between 2 and 9 mm, measured as the average of three diameters in longitudinal and transverse planes, or by considering the diameter of any follicle appearing circular in the scan. Alternatively, PCOM could also be defined by Ovarian volume (OV) > 10 cm^3^ in at least one ovary.

We divided the PCOS group into phenotypes according to the Rotterdam criteria. Phenotype A (PhA) includes women meeting all three diagnostic criteria: hyperandrogenism, ovulatory dysfunction, and PCOM. Phenotype B (PhB) meets the same criteria except for PCOM. Phenotype C (PhC), known as ovulatory PCOS, presents hyperandrogenism and PCOM without ovulatory dysfunction. Phenotype D (PhD) is a non-hyperandrogenic form, with oligo- or anovulation and PCOM, but with normal androgen levels. However, PhD was not considered in the present study since the sample was too small to analyze (*n* = 8) and consequently was not included.

The variables considered in this study were BMI (kg/m^2^), total cholesterol (mg/dL), HDL-cholesterol (mg/dL), non-HDL cholesterol (mg/dL), LDL-cholesterol (mg/dL), Apolipoprotein A (Apo A, mg/dL), Apolipoprotein B (Apo B, mg/dL), the Apo B/Apo A ratio, Lipoprotein A (Lp(a), mg/dL), triglycerides (mg/dL), fasting insulin (µU/mL), Insulin Growth Factor 1 (IGF-1, ng/mL), Homeostatic Model Assessment for Insulin Resistance (HOMA-IR), the glucose/fasting insulin ratio (G/I), log-transformed fasting insulin levels, and the Quantitative Insulin Sensitivity Check Index (QUICKI). Moreover, we considered the gonadotropins follicle-stimulating hormone (FSH, IU/mL) and Luteinizing Hormone (LH, IU/mL), as well as Androstenedione (A4, ng/mL), Sex Hormone-Binding Globulin (SHBG, nmol/L), dehydroepiandrosterone sulfate (DHEAS, ng/mL), and Free Testosterone (FT, pg/mL). Follicle count and ovarian volume were assessed via transvaginal ultrasound during the early follicular phase. Pelvic transvaginal ultrasound examinations were conducted using a VolusonS10 Expert device (GE HealthCare, Chicago, IL, USA) equipped with a microconvex intracavitary probe (RIC59) with an approximate frequency range of 5–9 MHz.

Ovarian volume was calculated by identifying three diameters and applying the ellipsoid formula π/6 × (length × width × thickness).

In oligomenorrheic patients, ultrasound was performed between 3–7 days counted from the beginning of menses. In amenorrheic women, ultrasound was performed at random timing. All the examinations were performed by an expert in reproductive gynecology and endocrinology with more than 10 years of expertise. Follicle count was performed on the long-axis plane of each ovary, and then the maximum value between the two ovaries was considered.

### 2.2. Data Analysis

All statistical analyses were performed using Jamovi (Version 2.7.37; The Jamovi Project, 2024—retrieved from https://www.jamovi.org, accessed on 25 July 2026). A Kolmogorov–Smirnov test indicated that all variables were non-normally distributed (*p* < 0.05). Therefore, comparisons between PCOS and control groups, as well as between phenotypes, were conducted using the Kruskal–Wallis test. Post-hoc comparisons were performed using the Dwass–Steel–Critchlow–Fligner (DSCF) test [21], followed by Bonferroni correction.

To assess the internal validation of the phenotype classification based on diagnostic criteria, Receiver Operating Characteristic (ROC) curve analyses were performed for the diagnostic criteria (follicle number, ovarian volume, and menstruations per year). These analyses were strictly intended to confirm the adherence and discrimination accuracy of the cohort assignment rather than an independent diagnostic evaluation. Moreover, ROC analyses were calculated for all the variables included in the study.

To address potential selection bias, a sensitivity analysis was conducted to compare demographic and baseline clinical characteristics between tested and untested subgroups for all variables showing a substantial proportion of missing data (approximately 50%).

## 3. Results

A total of 289 PCOS patients (85.25%) and 50 controls (14.75%) were included in the study. Among PCOS patients, 46.36% (*n* = 134) were classified as phenotype A (PhA), 14.88% (*n* = 43) as phenotype B (PhB), and 38.75% (*n* = 112) as phenotype C (PhC) (Table 1). All three PCOS groups were significantly younger than the control women. BMI differed significantly across phenotypes and controls (*p* < 0.001, corrected), with higher BMI values in PhA compared with PhC (*p* = 0.04, corrected) and controls (*p* < 0.001, corrected).

The number of menstruations per year was significantly lower in PhA and PhB compared with controls (*p* < 0.001). PhA and PhB also showed fewer menstruations than PhC, which did not differ from the control group (*p* = 0.112). Follicle number differed significantly among phenotypes, with PhA showing the highest median value and significantly higher counts than controls (*p* < 0.001). The histograms with the distribution of follicle number can be found in the Supplementary Materials (Figure S1). Ovarian volume was similar between PhA and PhC (*p* = 0.42), while PhB and controls showed comparably lower volumes (*p* = 0.52).

Regarding lipid parameters, PhA exhibited significantly lower HDL levels (*p* = 0.014, uncorrected) and higher triglyceride levels (*p* < 0.05, uncorrected) compared with controls.

Due to missing data for Apo A, Apo B, and Lp(a), which resulted from the fact that these dosages only began being obtained after the initiation of this study, we conducted a sensitivity analysis to evaluate potential selection bias. This analysis was performed under the assumption that the missing data mechanism for these parameters is Missing Not At Random (MNAR) across all phenotypes and controls. We found no statistically significant differences between the tested and untested subgroups regarding the other clinical and demographic variables, both within phenotypes and controls (*p* > 0.05, Bonferroni corrected). However, a significant difference in IGF-1 levels (*p* < 0.001, corrected) was observed between patients with and without available Apo A and Apo B data specifically within the PhA group (see Table S1, Supplementary Materials).

Several insulin-related parameters, ratios, and indices differed markedly between groups. Fasting insulin, log fasting insulin, QUICKI, HOMA-IR, and G/I levels were significantly higher in all three phenotypes compared with controls (Table A1), with a significant difference in HOMA_IR between PhA and PhC (*p* < 0.001). IGF-1 showed a small uncorrected difference (*p* < 0.02) without significant post-hoc comparisons.

**Figure 1:**
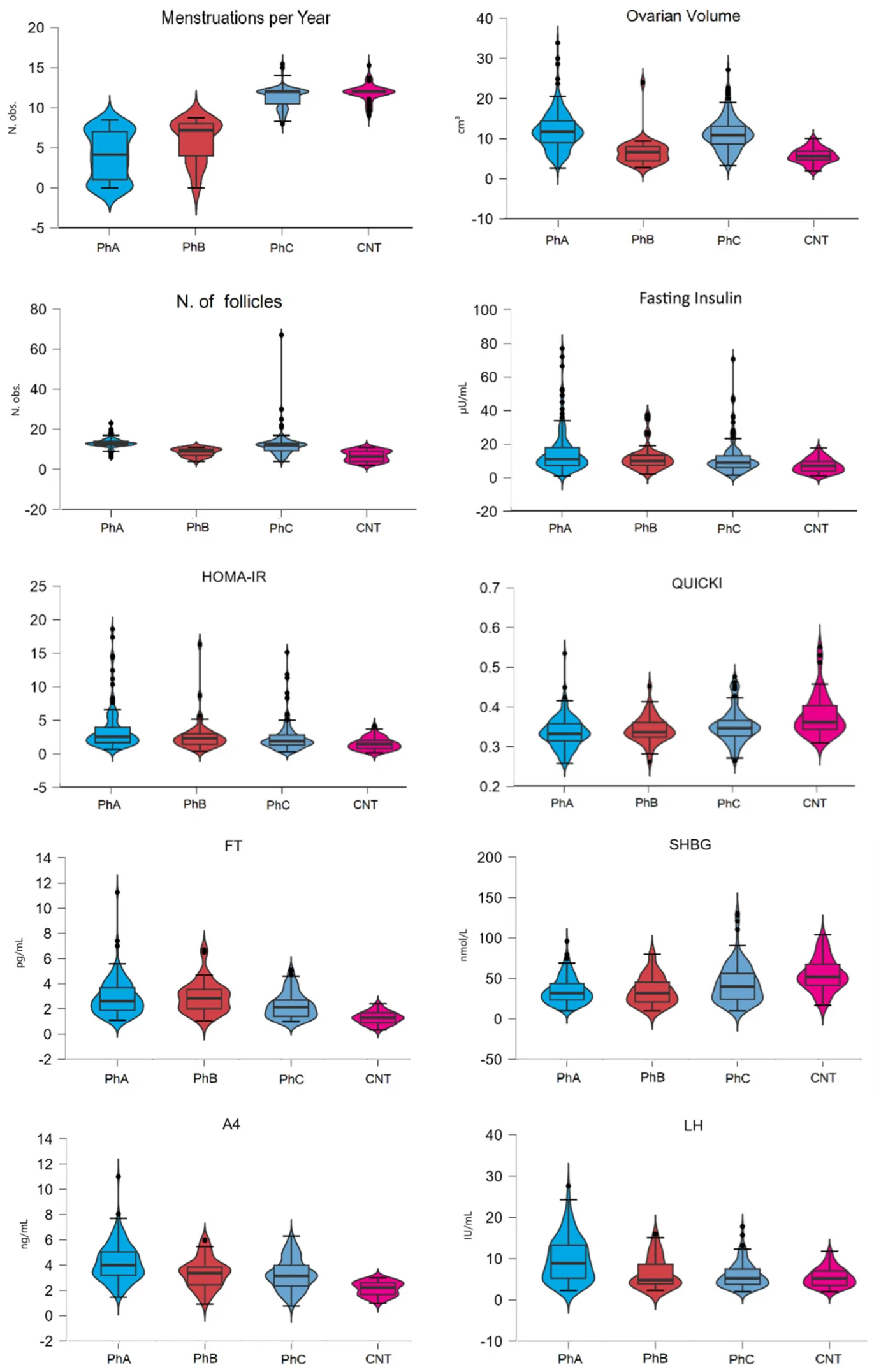
Between-group results. The figure depicts box-and-whisker plots with violin plots corresponding to between-group results for the variables Menstruation per year, ovarian volume, number of follicles, fasting insulin, HOMA-IR, QUICKI, FT, SHBG, A4, and LH (between-group significant results can be found in Table A1, Appendix A).

Similarly, among the clinical androgenic parameters, A4, SHBG, FT, and LH exhibited significant differences within the phenotypes and between patients and the control group, with *p*-values less than 0.001 (see Table 1; Figure 1). LH and A4 demonstrated notably higher and significant differences when comparing PhA and PhC to the control group. Additionally, SHBG and FT showed significant differences among all three phenotypes in comparison to the control group (*p* < 0.001; see Table A1).

**Figure 2:**
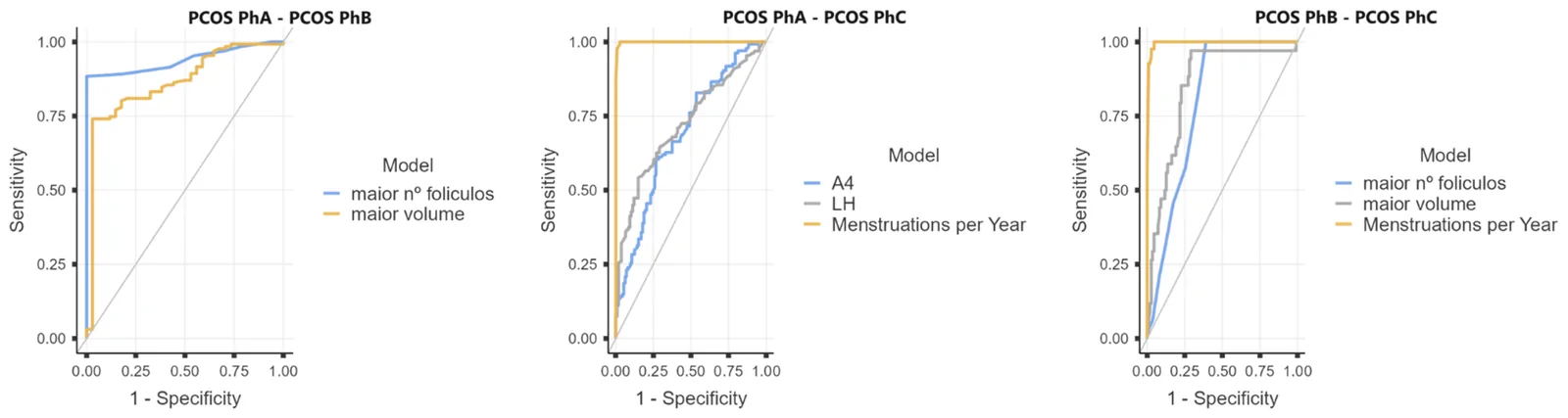
ROC Results.

A similar pattern was observed for HOMA-IR, QUICKI, the glucose/insulin ratio (G/I), and log-transformed fasting insulin (Figure 2), with all phenotypes showing altered values compared with controls and PhA exhibiting higher insulin resistance than PhC (*p* < 0.001; Table A1).

Despite the initial variations observed for metabolic parameters, the unadjusted Kruskal–Wallis test highlighted significant differences in age and BMI among the groups. To account for these confounding variables, a multivariable robust regression analysis was performed (Table 2). For the lipid profile biomarkers, the model revealed no significant main effect of the group after controlling for age and BMI; instead, a significant effect of both covariates was observed for most of these parameters. Conversely, among the insulin-related biomarkers, a significant phenotype effect persisted for the G/I ratio (*p* < 0.01), Log Fasting Insulin (*p* < 0.05), and QUICKI (*p* < 0.005). Concurrently, a substantial independent effect of BMI was observed for these indices, as well as for HOMA-IR (*p* < 0.001).

All variables were subsequently included in ROC analyses. Follicle number showed strong discriminative power between PhA and PhB (AUC = 0.939; 95% CI: 0.904–0.974; SE = 0.017; cutoff = 11.5; *p* < 0.001) and between PhB and PhC (AUC = 0.795; 95% CI: 0.725–0.866; SE = 0.035; cutoff = 11.5; *p* < 0.001).

Similarly, ovarian volume significantly discriminated between PhA and PhB (AUC = 0.864; 95% CI: 0.795–0.932; SE = 0.035; cutoff = 9.45; *p* < 0.001) and between PhB and PhC (AUC = 0.849; 95% CI: 0.775–0.922; SE = 0.037; cutoff = 9.39; *p* < 0.001). Menstruations per year also showed excellent discriminative ability between PhA and PhC (AUC = 0.999; 95% CI: 0.997–1.000; SE = 0.001; cutoff = 8.52; *p* < 0.001) and between PhB and PhC (AUC = 0.996; 95% CI: 0.990–1.000; SE = 0.002; cutoff = 8.15; *p* < 0.001).

Regarding androgens, A4 showed moderate discriminative power between PhA and PhC (AUC = 0.690; 95% CI: 0.624–0.756; SE = 0.0336; cutoff = 3.635; *p* < 0.001). LH also showed moderate discriminative power between PhA and PhC (AUC = 0.721; 95% CI: 0.657–0.785; SE = 0.721; cutoff = 8.55; *p* < 0.001).

Significant ROC values for phenotype–control comparisons are reported in Table A2.

Moreover, to explore the association between ovarian morphology, androgens, and metabolic variables, we performed a series of Spearman’s rho correlations. All the considered variables were inserted in a 9 × 15 correlation matrix, and the results were corrected for False Discovery Rate (FDR), applying the Benjamini–Hochberg method [22] for the whole sample (Table S3, Supplementary Materials). Then, partial Spearman’s correlations, with age and BMI as covariates, were calculated.

In PhA, we observed significant positive associations between FT and non-HDL cholesterol, LDL, fasting insulin, and log fasting insulin (*p* < 0.05 FDR corr.) and negative associations with G/I and QUICKI (*p* < 0.05 FDR corr.). A similar pattern was observed for SHBG that showed significant negative correlations with triglycerides, fasting and log fasting insulin, HOMA-IR, and QUICKI and positive associations with HDL that were not completely affected by the effects of age and BMI.

Interestingly, in PhB, follicle number showed a negative FDR-corrected correlation with fasting insulin and log fasting insulin (*p* < 0.005). These correlations were not affected by the covariates but showed a weaker association (*p* < 0.05).

PhC showed a pattern similar to that observed in PhA. Indeed, FT showed a significant positive association with HOMA-IR and a negative association with QUICKI. Despite SHBG and FT showing robust correlations with the insulin-related parameters, this effect was dependent on the effect of covariates (age and BMI), with no significant results for the partial correlation (Table S2 Supplementary Results).

## 4. Discussion

In this retrospective cross-sectional study, we compared the three PCOS phenotypes and identified differences in follicle number, ovarian volume, insulin-related biomarkers, androgens, and LH and FSH. Nearly half of the PCOS patients belonged to phenotype A (46.36%), followed by the ovulatory phenotype C, while phenotype B represented only 15% of the sample. This partially aligns with previously published evidence [23], which reported that phenotypes A and B together accounted for two-thirds of the total sample.

Specifically, we observed that follicle number, but not ovarian volume, showed a negative correlation with insulin resistance in phenotype B.

Our data suggest that correlations between insulin resistance markers (fasting insulin, HOMA-IR, QUICKI) and signs of polycystic ovarian morphology tend to be negative. This indicates that the hyperinsulinemia characteristic of PCOS/PCOM may actually oppose the formation of the classic “string of pearls” appearance described in the Rotterdam criteria. This correlation was significant only in phenotype B, which may help explain the coexistence of phenotypes A and B: in phenotype B, higher insulin resistance may inhibit the development of multiple follicles, whereas in phenotype A, lower insulin resistance may not suppress follicular proliferation. This suggests that insulin resistance may not only contribute to androgen excess, but also modulate ovarian morphology itself. The presence of multifollicular or polycystic morphology may therefore result from differences in ovarian responsiveness to hyperinsulinemia, implying an interaction between insulin signaling and follicular dynamics.

Overall, our results highlight the relevance of ovarian dysmorphology in PCOS and its association with hyperinsulinemia and insulin resistance. As recent guidelines emphasize the involvement of multiple endocrine systems in PCOS, ovarian morphology remains clinically important for phenotype discrimination and personalized treatment planning.

Although follicles are not pathological cysts, our findings indicate that follicle number can discriminate among phenotypes and is associated with insulin-related alterations, one of the most clinically relevant aspects of PCOS. New insights may emerge from advanced imaging techniques, including high-resolution ultrasound and magnetic resonance imaging (MRI). Follicle distribution patterns appear more complex than the classic “string of pearls” sign [24]. Mills [25] recently reported that women with a string of pearls pattern experience more menstrual irregularities than those with a diffuse follicular distribution. Future studies using advanced image-processing techniques may help clarify the relationship between ovarian morphology and metabolic dysfunction.

Dysmorphic ovaries have also been reported to be common in obese women of reproductive age and are associated with insulin resistance [26]. This condition can improve with adherence to hypocaloric dietary regimens, such as the Mediterranean diet, suggesting a possible mechanistic link between PCOS, PCOM, and insulin resistance.

Since the first description of PCOS-related insulin resistance in 1980 [27], the association between insulin resistance, hyperinsulinemia, and PCOS has been widely documented. A systematic review by Cassar et al. [28] confirmed that insulin resistance is a key feature of PCOS. A 27% reduction in insulin sensitivity among women with PCOS indicates that they require higher insulin levels to maintain normal glucose homeostasis. Importantly, this intrinsic insulin resistance occurs independently of body mass index (BMI), meaning that even lean women with PCOS may exhibit significant metabolic impairment. However, in our study, we found that BMI is not independent of insulin resistance (Table 2).

Cut-off values for HOMA-IR remain inconsistent across studies. Although the European Group for the Study of Insulin Resistance recommends a threshold above 2.0 [29], other studies have reported cut-offs ranging from 1.55 to ≥3.8 [30,31]. In our cohort, insulin resistance assessed by HOMA-IR exceeded the clinical threshold in phenotypes A (2.56) and B (2.32), but not in phenotype C (1.90), which showed a near-threshold median value. ROC analyses revealed that the significant (*p* < 0.001) clinical cut-offs for distinguishing PhA vs controls and PhB vs controls were slightly lower than the recommended values. Interestingly, HOMA-IR and fasting insulin lacked discriminative power among the phenotypes.

We also found globally elevated IGF-1 levels, although no significant differences emerged between phenotypes. Hyperinsulinemia in PCOS inhibits hepatic production of IGF-1–binding protein, resulting in increased circulating IGF-1 [32,33]. Elevated IGF-1 further stimulates ovarian androgen production [34]. Insulin enhances androgen synthesis by ovarian theca cells both directly and indirectly, and increased IGF-1, due to reduced binding protein, is one mechanism linking hyperinsulinemia to androgen excess. No significant correlations were observed between IGF-1 and ovarian volume or follicle number.

Moreover, elevated free testosterone levels have been independently linked to impaired insulin action in both glucose and lipid metabolism in PCOS [35]. In contrast to other studies, even though PhB showed the highest median values of FT, we found no significant differences among the phenotypes [35]. However, all phenotypes in this study presented significantly higher FT levels than controls, consistent with Gihad Falh et al. [36]. Our results showed that in PhA, FT was strongly associated with insulin-related biomarkers that were partially independent of age and BMI, as well as with LDL and non-HDL cholesterol. A similar result was observed for PhC.

Emerging evidence suggests serum A4 could be a valuable marker for identifying insulin resistance (IR) and assessing metabolic risk in women with PCOS [37]. Our results indicate that all PCOS phenotypes had significantly higher A4 levels compared to controls. Notably, we found that individuals with the A phenotype (PhA) exhibited significantly higher A4 levels than those with the C phenotype (PhC). In fact, A4 was the only androgen analyzed that exhibited significant discriminative power among the PCOS phenotypes in our findings. A4 showed moderate discriminative power between PhA and PhC, with a cut-off value of 3.6. Tosi et al. [35] also reported elevated A4 levels in classic PCOS compared to ovulatory PCOS. These findings align with the understanding that PhA is associated with an increased risk of adverse metabolic conditions, such as insulin resistance (IR). It is generally believed that IR is a key factor contributing to the development of metabolic syndrome. Additionally, our results showed that PhA has significantly higher HOMA-IR values than PhC.

Increased DHEAS concentrations have been reported at a lower prevalence in A phenotype PCOS patients, who generally have the highest androgen levels, versus non-classic PCOS phenotypes [38]. Approximately 33% of non-classic PCOS patients were reported by Carmina and Longo to have elevated DHEAS, with higher serum DHEAS levels in PhC compared with PhA. However, our study found no significant differences in DHEAS levels among PCOS phenotypes or when compared to controls. These findings align with those of Boucher et al. [39], who reported no significant differences in DHEAS among PCOS phenotypes.

Low levels of SHBG lead to sustained increases in the circulating free androgen concentration. This rise reduces the hypothalamus’s sensitivity to LH pulses. As a result, excessive gonadotropin-releasing hormone (GnRH) release stimulates the pituitary gland to release more LH [40]. Indeed, in the present study, PhA and PhB showed significantly lower SHBG than controls, with PhC presenting the highest levels of SHBG among the phenotypes. However, only PhA showed higher LH than PhC and controls, not PhB. In Phenotype C, SHBG showed a significant negative association with insulin biomarkers such as fasting insulin and HOMA-IR (positive with QUICKI), but dependent on the effect of age and BMI.

These elevated LH levels also stimulate theca cells in the ovaries to produce more androgens while simultaneously inhibiting FSH’s action on granulosa cells, which are essential for follicle maturation [41]. As a result, this leads to anovulation or irregular ovulation, accompanied by menstrual irregularities and infertility. While a typical menstrual cycle relies on the anterior pituitary gland to release balanced levels of FSH and LH to stimulate the development of ovarian follicles and ovulation, this regulatory mechanism becomes impaired in individuals with PCOS. FSH levels are often reported to be low or normal in PCOS [41]. Indeed, our findings also indicate that there are no significant differences in FSH levels among different phenotypes or when compared to controls. However, the relative deficiency of FSH compared to elevated LH levels disrupts normal follicular dynamics. This imbalance causes improper follicle maturation and ovulation, leading to multiple immature follicles that may resemble cysts on ultrasound [42].

Follicle number and ovarian volume differed significantly among phenotypes. Phenotype B showed the lowest median values (9 follicles; 6.68 cm^3^), while phenotypes A and C showed higher values (13 follicles and 11.7 cm^3^; 12 follicles and 10.8 cm^3^, respectively). The presence of at least 12 antral follicles remains a principal Rotterdam criterion for distinguishing PCOM from non-PCOM patients [24,43]. With advances in ultrasound imaging, a threshold of ≥26 antral follicles has been proposed [44], especially because it became possible to assess the number of follicles in the entire volume of the ovaries instead of only in the equatorial plane. Although phenotype B is typically not associated with PCOM, we found a higher number of antral follicles compared with controls. The significant cut-off distinguishing PhA vs PhB and PhB vs PhC was 11.5, similar to the Rotterdam threshold. Hong et al. [11] reported mean follicle counts of 13.9 in PCOM and 6.5 in non-PCOM patients, and, consistent with our findings, identified follicle number as a significant predictor of insulin resistance assessed by QUICKI. Our finding partially confirmed this result, showing that the value of fasting insulin observed in PhB was negatively related to follicle number, which was significantly lower than in PhA and PhC.

In our study, ovarian volume and follicle number were effective markers for distinguishing among the three phenotypes and between the patients and controls, consistent with Jonard et al. [43].

We found that an ovarian volume cut-off of 9.5 cm^3^ discriminated PhA vs PhB and PhB vs PhC, in line with the Rotterdam criteria. Similarly, Villa et al. [45] reported an average ovarian volume of 9.6 cm^3^ in adolescents with PCOS and a BMI of 25.7.

Phenotype A showed significantly lower HDL and higher triglyceride levels compared with controls. However, no differences in lipid parameters were observed among the PCOS phenotypes, and none of the lipid biomarkers discriminated between phenotypes. Mild alterations in HDL and triglycerides are common in PCOS, and phenotype A typically exhibits the worst metabolic profile [46]. Carmina et al. [14] reported similar findings in PCOS patients from Sicily. Our BMI values (<27.4) were comparable to those reported in Sicilian PCOS patients, suggesting similar lifestyle and dietary patterns between the two Mediterranean populations. Despite this, we did not observe significant alterations in total cholesterol, triglycerides, or HDL across phenotypes, consistent with findings from other Mediterranean cohorts [47,48].

Interestingly, Lp(a) did not show significant between-group differences or associations with follicle number. This does not agree with a recent meta-analysis by Masson et al. [49] that found that women with PCOS have higher Lp(a) levels than controls, regardless of BMI. IGF-1 suppresses hepatic synthesis of Lp(a), which consists of an LDL-like particle bound to apolipoprotein(a) and is an independent genetic risk factor for atherosclerotic cardiovascular disease. Laron [50] hypothesized that IGF-1-mediated reduction in Lp(a) may be linked to its insulin-suppressing effects.

Evidence from different geographic regions indicates that the metabolic features of PCOS are strongly influenced by ethnic background and behavioral factors. In Mediterranean countries, obesity, type 2 diabetes, and dyslipidemia appear to be less common among PCOS patients [51].

Several limitations must be acknowledged. The phenotype sample sizes were heterogeneous. Although the sample may be representative of the Portuguese population, the study was not multicentric or multiethnic.

While the diagnostic classification was based on consistent clinical criteria, androgen measurements were conducted using CLIA methods instead of Liquid Chromatography–Tandem Mass Spectrometry (LC–MS/MS). The substantial amount of missing data for specific lipid biomarkers (e.g., Lp(a), ApoA, and ApoB) represents a limitation, which was assessed by applying a sensitivity analysis showing an effect of missing data in ApoA and ApoB and in PhA for IGF-1. For this reason, the results related to IGF-1, Lp(a), ApoA, and ApoB need to be taken cautiously. Given that, we cannot entirely rule out selection bias, and the smaller sample size in these specific subgroups may have limited the statistical power to detect subtler correlations.

We need to acknowledge that the present study did not take into account the role played by Anti-Mullerian Hormone (AMH) in PCOS and follicle count.

## 5. Conclusions

The present study suggests a complex interplay among ovarian morphology, androgen, and metabolic patterns in the different PCOS phenotypes. This is in agreement with the new framework, Polyendocrine Metabolic Ovarian Syndrome, which highlights the interplay between different endocrine and metabolic systems that are affected in PCOS/PMOS.

We have demonstrated that alterations in ovarian morphology play a relevant role. We have also identified the reason why there are 2 different phenotypes discriminated only by ovarian morphology. Although both have significant levels of insulin resistance, in phenotype B the ovaries are more sensitive to its negative morphological influence.

Finally, our analysis allowed the determination of cutoff values for some insulin resistance parameters and for the number of ovarian follicles and ovarian volumes in the PCOS phenotypes.

## Figures and Tables

**Table 1:** Comparisons between PCOS phenotypes and controls for each variable considered.

Variables	Groups	N	Median	IQR	H (df = 3)	*p* Value	ε^2^
AGE	PhA	134	22	8	26.89	<0.001 *	0.079
(years)	PhB	43	19	7.5			
	PhC	112	22	9.25			
	CNT	50	28	11.25			
BMI	PhA	133	27.476	11.10	22.9	<0.001 *	0.068
(kg/m^2^)	PhB	43	23.398	8.934			
	PhC	108	24.925	9.452			
	CNT	49	22.145	4.653			
Menstruations per Year	PhA	130	4.143	5.977	258.05	<0.001 *	0.784
	PhB	41	7.2	4			
	PhC	109	12	1.5			
	CNT	50	12	0			
Cholesterol	PhA	126	176	48.25	1.93	0.588	0.006
(mg/dL)	PhB	33	178	47			
	PhC	105	171	40			
	CNT	44	168	46			
HDL	PhA	122	51	16	1.69	0.014	0.035
(mg/dL)	PhB	32	52.5	13			
	PhC	102	51	15.75			
	CNT	42	56	11.75			
Non-HDL cholesterol	PhA	122	120	54	3.55	0.315	0.011
(mg/dL)	PhB	32	121.5	38.75			
	PhC	102	111	44			
	CNT	42	109	37.25			
LDL	PhA	121	104.6	42.6	1.69	0.639	0.005
(mg/dL)	PhB	31	101.4	33.3			
	PhC	99	97.6	39.5			
	CNT	41	98.8	38.4			
Apo A	PhA	95	141	28	4.01	0.260	0.019
(mg/dL)	PhB	19	150	37.5			
	PhC	65	146	32			
	CNT	23	148	31			
Apo B	PhA	95	83	27.5	6.45	0.092	0.032
(mg/dL)	PhB	19	79	29			
	PhC	64	7.5	32.25			
	CNT	22	69.5	25.25			
Apo B/Apo A	PhA	95	0.553	0.236	8.94	0.030	0.044
	PhB	19	0.535	0.242			
	PhC	64	0.484	0.205			
	CNT	22	0.48	0.129			
Lp(a)	PhA	84	13.1	27.1	1.57	0.666	0.008
(mg/dL)	PhB	15	12	48.4			
	PhC	61	11.7	23.4			
	CNT	18	12	32.5			
Triglycerides	PhA	125	79	56	1.48	0.015	0.034
(mg/dL)	PhB	33	61	32			
	PhC	101	68	33			
	CNT	42	64	32.25			
Fasting Insulin	PhA	129	11	1.7	29.98	<0.001 *	0.091
(µU/mL)	PhB	41	10	6			
	PhC	110	9.05	7.125			
	CNT	47	7	5.75			
HOMA-IR	PhA	120	2.559	2.287	38.02	<0.001 *	0.119
	PhB	41	2.320	1.545			
	PhC	110	1.898	1.502			
	CNT	47	1.476	1.235			
G/I	PhA	128	7.966	6.162	29.41	<0.001 *	0.090
	PhB	41	8.3	4.120			
	PhC	110	9.660	5.964			
	CNT	47	12.857	13.171			
log fasting Insulin	PhA	128	1.041	0.387	27.35	<0.001 *	0.084
	PhB	41	1	0.257			
	PhC	110	0.957	0.339			
	CNT	45	0.845	0.389			
QUICKI	PhA	128	0.332	0.043	31.79	<0.001 *	0.097
	PhB	41	0.336	0.036			
	PhC	110	0.346	0.039			
	CNT	48	0.362	0.059			
IGF1	PhA	121	264	158	1.98	0.012	0.036
(ng/mL)	PhB	39	360	225.25			
	PhC	97	292	241			
	CNT	43	247	149			
ovarian volume	PhA	131	11.760	5.455	103.97	<0.001 *	0.329
(cm3)	PhB	34	6.685	3.527			
	PhC	110	1.855	4.462			
	CNT	42	5.570	2.217			
Follicle number	PhA	129	13	2	129.97	<0.001 *	0.415
	PhB	33	9	3			
	PhC	110	12	3.75			
	CNT	42	6.5	5			
A4	PhA	134	3.99	1.84	91.54	<0.001 *	0.271
ng/mL	PhB	43	3.37	1.395			
	PhC	112	3.15	1.6425			
	CNT	49	2.23	0.92			
DHEAS	PhA	133	2260	1617	15.73	0.001	0.047
ng/mL	PhB	43	2420	1810			
	PhC	110	2550	1411			
	CNT	49	1771	1380			
SHBG	PhA	130	31.8	2.3	36.18	<0.001 *	0.110
nmol/mL	PhB	39	31.6	24.6			
	PhC	110	39.75	31.925			
	CNT	49	52	26.1			
FT	PhA	128	2.6	1.7825	79.98	<0.001 *	0.263
pg/mL	PhB	39	2.84	1.54			
	PhC	90	2.13	1.2875			
	CNT	48	1.3	0.785			
FSH	PhA	131	5.9	2.05	7.67	0.053	0.023
IU/mL	PhB	40	5.3	1.882			
	PhC	107	5.62	1.925			
	CNT	49	6.3	1.92			
LH	PhA	131	8.9	8	47.12	<0.001 *	0.144
IU/mL	PhB	40	4.815	4.775			
	PhC	107	5.23	3.7			
	CNT	49	5.2	3.5			

Abbreviations: BMI = Body Mass Index; LDL = Low Density Lipoprotein; HOMA-IR = Homeostatic Model Assessment of Insulin Resistance; G/I = Glucose Insulin Ratio; IGF 1 = Insulin-like growth Factor 1; ApoA = Apolipoprotein A; Apo B = Apolipoprotein B; Lp(a) = Lipoprotein a; HDL = High Density Lipoprotein; QUICKI = Quantitative insulin sensitivity check index; A4 = Androstenedione; DHEAS = Dehydroepiandrosterone sulfate; SHBG =Sex Hormone-Binding Globulin; FT = Free testosterone; FSH = Follicle-stimulating hormone; LH = Luteinizing hormone; PCOS = Polycystic Ovary Syndrome; CNT= controls; IQR = interquartile range; df = degree of freedom; N = number; ε^2^ denotes the effect size.; * denotes Bonferroni’s corrected values.

**Table 2:** Multivariable analysis of metabolic outcomes adjusted for age and body mass index across phenotype groups.

Metabolic Outcome	Group Effect (Phenotype)	Covariate AGE	Covariate BMI
Cholesterol	F = 2.35, *p* = 0.072	F = 29.88, *p* < 0.001	F = 2.88, *p* = 0.09
HDL	F = 0.59, *p* = 0.619	F = 0.029, *p* = 0.863	F = 46.29, *p* < 0.001
Non-HDL Cholesterol	F = 2.48, *p* = 0.06	F = 29.76, *p* < 0.001	F = 18.721, *p* < 0.001
LDL	F = 1.64, *p* = 0.178	F = 2.221, *p* < 0.001	F = 8.29, *p* = 0.0049
Apo A	F = 0.10, *p* = 0.956	F = 1.185, *p* = 0.277	F = 11.46, *p* < 0.001
Apo B	F = 3.209, *p* = 0.024	F = 28.789, *p* < 0.001	F = 8.977, *p* < 0.005
Apo A/ApoB	F = 2.031, *p* = 0.111	F = 14.882, *p* < 0.001	F = 19.955, *p* < 0.001
Lp(a)	F = 1.221, *p* = 0.304	F = 1.289, *p* = 0.258	F = 0.494, *p* = 0.483
Triglycerides	F = 2.584, *p* = 0.053	F = 13.292, *p* < 0.001	F = 26.081, *p* < 0.001
Fasting Insulin	F = 1.558, *p* = 0.2	F = 0.018, *p* = 0.891	F = 108.061, *p* < 0.001
HOMA-IR	F = 2.627, *p* = 0.050	F = 1.797, *p* = 0.181	F = 85.708, *p* < 0.001
G/I	F = 3.875, *p* < 0.01	F = 4.114, *p* < 0.05	F = 52.791, *p* < 0.001
Log Fasting Insulin	F = 2.744, *p* < 0.05	F = 1.785, *p* = 0.182	F = 144.241, *p* < 0.001
QUICKI	F = 5.475, *p* < 0.005	F = 0.61, *p* = 0.435	F = 97.831, *p* < 0.001
IGF-1	F = 2.004, *p*= 0.113	F = 81.628, *p* < 0.001	F = 2.729, *p* = 0.099

Note: Data are presented as F-statistics and corresponding *p*-values derived from General Linear Models with robust standard errors (HC3 estimation method). LDL = Low Density Lipoprotein; HOMA-IR = Homeostatic Model Assessment of Insulin Resistance; G/I = Glucose Insulin Ratio; IGF 1 = Insulin-like growth Factor 1; ApoA = Apolipoprotein A; Apo B = Apolipoprotein B; Lp(a) = Lipoprotein a; HDL = High Density Lipoprotein; QUICKI = Quantitative insulin sensitivity check index. BMI: Body Mass Index.

**Figure A1:**
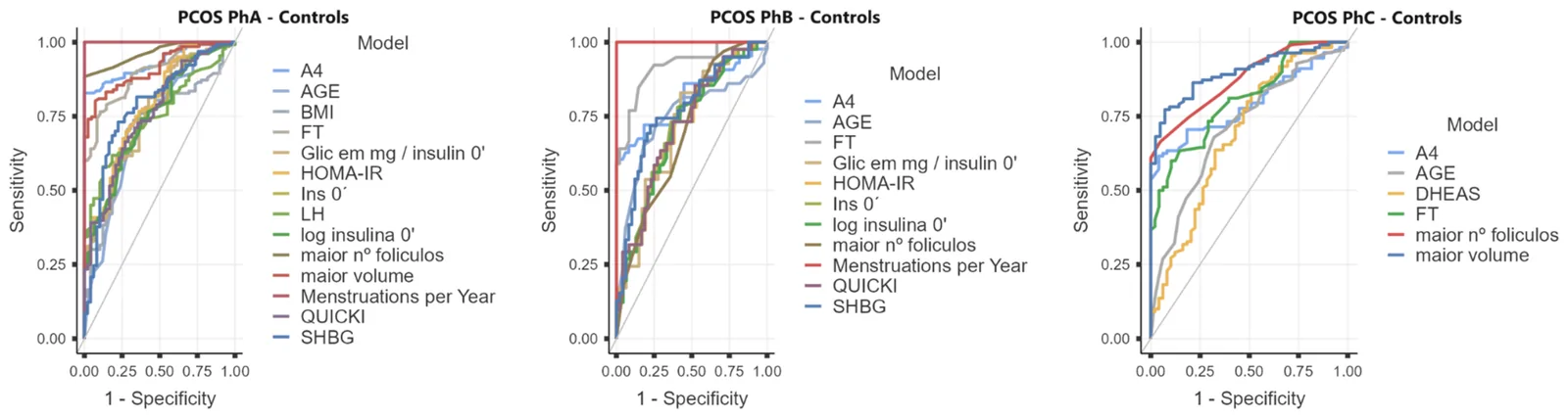
ROC curve results.

## Data Availability

The data are available from the corresponding author on reasonable request.

## Supplementary Materials

The following supporting information can be downloaded at: https://www.mdpi.com/article/10.3390/jpm16100512/s1, Figure S1: Histograms and detailed reports of the distributions of the follicle number for each group; Table S1:Sensitivity Analysis results for the missing values of Apo A, Apo B, and Lp(a); Table S2: Spearman Correlation and Partial Spearman correlation results (BMI and AGE as covariates); Table S3: False Discovery Rate-corrected values and 95% Confidence intervals.

## Appendix A

**Table A1:** Multiple comparisons results.

	PhB	PhC	CNT		PhB	PhC	CNT
Age				HOMA-IR			
PhA	0.291	0.989	<0.001	PhA	0.244	<0.001	<0.001
PhB		0.526	<0.001	PhB		0.644	0.003
PhC			<0.001	PhC			0.021
BMI				G/I			
PhA	0.078	0.032	<0.001	PhA	0.718	0.033	<0.001
PhB		0.982	0.339	PhB		0.688	0.002
PhC			0.061	PhC			0.011
Menstruations per year				Log Fasting Insulin			
PhA	0.002	<0.001	<0.001	PhA	0.617	0.015	<0.001
PhB		<0.001	<0.001	PhB		0.656	0.005
PhC			0.112	PhC			0.032
HDL				QUICKI			
PhA	0.514	0.734	0.014	PhA	0.539	0.007	<0.001
PhB		0.874	0.354	PhB		0.644	0.002
PhC			0.060	PhC			0.013
ApoA/ApoB				IGF 1			
PhA	0.940	0.091	0.055	PhA	0.139	0.143	0.742
PhB		0.811	0.592	PhB		0.950	0.068
PhC			0.979	PhC			0.064
Triglycerides				Ovary Volume			
PhA	0.236	0.085	0.042	PhA	<0.001	0.420	<0.001
PhB		0.991	0.887	PhB		<0.001	0.526
PhC			0.797	PhC			<0.001
Fasting Insulin				N. of Follicles			
PhA	0.650	0.018	<0.001	PhA	<0.001	<0.001	<0.001
PhB		0.656	0.002	PhB		<0.001	0.017
PhC			0.011	PhC			<0.001
LH				SHBG			
PhA	0.001	<0.001	<0.001	PhA	0.999	0.028	<0.001
PhB		0.990	0.919	PhB		0.174	<0.001
PhC			0.966	PhC			0.007
A4				FT			
PhA	0.008	<0.001	<0.001	PhA	0.973	0.002	<0.001
PhB		0.844	<0.001	PhB		0.015	<0.001
PhC			<0.001	PhC			<0.001

**Table A2:** ROC analysis results for the models comparing phenotypes (A, B, and C) and controls (*p* < 0.001).

PhC vs. CNT	AUC	Std. Error	Youden’s Index	95% CI (Lower-Upper)	Cut-Off
AGE	0.712	0.0430	0.35857	0.627–0.796	25.5
Ovary volume	0.898	0.0245	0.7013	0.850–0.946	8.345
FT	0.807	0.0361	0.496	0.736–0.878	1.825
A4	0.792	0.0342	0.5753	0.725–0.859	2.955
DHEAS	0.692	0.0472	0.3171	0.600–0.784	1720
Follicle number	0.876	0.0269	0.616	0.823–0.928	1.5
PhB vs. CNT	AUC	Std. Error	Youden’s Index	95% CI (Lower-Upper)	Cut-off
AGE	0.743	0.0556	0.471	0.635–0.852	21.5
Fasting Insulin	0.721	0.0542	0.376	0.614–0.827	7.15
HOMA-IR	0.713	0.0548	0.3487	0.605–0.820	1.586
G/I	0.719	0.0545	0.404	0.612–0.825	12.227
Log Fasting insulin	0.708	0.0559	0.3583	0.599–0.818	0.854
QUICKI	0.718	0.0540	0.3567	0.613–0.824	0.356
FT	0.920	0.0297	0.700	0.861–0.978	1.75
A4	0.816	0.0477	0.6047	0.722–0.909	3.105
SHBG	0.768	0.0518	0.493	0.667–0.870	4.15
Follicle number	0.698	0.0593	0.297	0.582–0.814	5.5
PhA vs. CNT	AUC	Std. Error	Youden’s Index	95% CI (Lower-Upper)	Cut-off
AGE	0.717	0.0432	0.336	0.632–0.801	26.5
BMI	0.714	0.0411	0.4211	0.633–0.794	24.25
Fasting Insulin	0.755	0.0393	0.3784	0.678–0.832	9.85
HOMA-IR	0.785	0.0387	0.423	0.710–0.861	1.904
G/I	0.760	0.0396	0.402	0.682–0.837	8.60
log Fasting Insulin	0.746	0.0401	0.3727	0.668–0.825	0.993
QUICKI	0.757	0.0390	0.3932	0.681–0.834	0.346
Ovary volume	0.911	0.0211	0.7301	0.870–0.952	8.25
Follicle number	0.964	0.0118	0.884	0.941–0.987	11.5
FT	0.898	0.0228	0.661	0.853–0.943	1.845
A4	0.930	0.0176	0.828	0.896–0.965	3.01
SHBG	0.784	0.0390	0.5063	0.707–0.860	41.05
LH	0.749	0.0361	0.4577	0.678–0.820	7.75
PhA vs. PhC	AUC	Std. Error	Youden’s Index	95% CI (Lower-Upper)	Cut-off
Menstruations per Year	0.999	0.00083	0.972	0.997–1.000	8.52
A4	0.690	0.0336	0.3366	0.624–0.756	3.635
LH	0.721	0.721	0.3925	0.657–0.785	8.55
PhA vs. PhB	AUC	Std. Error	Youden’s Index	95% CI (Lower-Upper)	Cut-off
Ovary volume	0.864	0.0350	0.7110	0.795–0.932	9.455
Follicle number	0.939	0.0177	0.884	0.904–0.974	11.5
PhB vs. PhC	AUC	Std. Error	Youden’s Index	95% CI (Lower-Upper)	Cut-off
Menstruations per Year	0.996	0.00285	0.954	0.990–1.000	8.86
Ovary volume	0.849	0.03740	0.67968	0.775–0.922	9.39
Follicle number	0.795	0.03586	0.609	0.725–0.866	11.5
