# Supplementary material for: The Relevance of Follicle Number and Androgens in Metabolic Alterations in Polycystic Ovary Syndrome: A Cross-Sectional Study
Source: J Pers Med. 2026 Sep 30;16(10):512. doi: 10.3390/jpm16100512 (PMC13706015; doi:10.3390/jpm16100512)
Supplement: Supplementary file 1 [file jpm-16-00512-s001.zip › jpm-4494737-supplementary.pdf]

Supplementary Table S1. Sensitivity Analysis results for the missing values of Apo A, Apo B, and Lp(a).

Ph A	APO A			APO B			LpA		
**Variables**	**t test**	**df**	**p-value**	**t test**	**df**	**p-value**	**t test**	**df**	**p-value**
**AGE**	.232	132	.817	.232	132	.817	-.282	132	.778
**BMI**	2.551	131	.012	2.551	131	.012	-1.477	131	.142
**DHEAS**	-.311	131	.756	-.311	131	.756	-.940	131	.349
**SHBG**	-3.116	128	.002	-3.116	128	.002	2.002	128	.047
**FSH**	2.788	129	.006	2.788	129	.006	-2.413	129	.017
**LH**	.004	129	.996	.004	129	.996	-.618	129	.537
**A4**	1.527	132	.129	1.527	132	.129	-3.072	132	.003
**Cholesterol**	-.700	124	.485	-.700	124	.485	.153	124	.879
**HDL**	-1.249	120	.214	-1.249	120	.214	-.092	120	.927
**Non-HDL cholesterol**	-.422	120	.673	-.422	120	.673	.248	120	.804
**LDL**	-.085	119	.932	-.085	119	.932	-.235	119	.814
**Triglycerides**	-1.140	123	.256	-1.140	123	.256	1.409	123	.161
**Fasting Insulin**	.913	127	.363	.913	127	.363	-1.077	127	.283
**HOMA-IR**	.994	118	.322	.994	118	.322	-.938	118	.350
**QUICKI**	.239	126	.811	.239	126	.811	-.329	126	.742
**log fasting insulin**	.104	126	.917	.104	126	.917	-.260	126	.795
**Ovarian volume**	1.043	129	.299	1.043	129	.299	-.467	129	.641
**Follicle number**	2.022	127	.045	2.022	127	.045	-2.176	127	.031
**Menstruations per Year**	.589	128	.556	.589	128	.556	.389	128	.697
**G/I**	.814	126	.417	.814	126	.417	-.592	126	.555
**IGF1**	**-3.626**	**119**	**<.001**	**-3.626**	**119**	**<.001**	2.570	119	.011
**PhB**									
	**APO A**			**APO B**			**LpA**		
**Variables**	t test	df	p-value	t test	df	p-value	t test	df	p-value
**AGE**	.121	41	.904	.121	41	.904	-.268	41	.790
**BMI**	.149	41	.882	.149	41	.882	.020	41	.984
**DHEAS**	.026	41	.979	.026	41	.979	.952	41	.347

**SHBG**	-.629	37	.533	-.629	37	.533	-.057	37	.954
**FSH**	1.588	38	.120	1.588	38	.120	-.989	38	.329
**LH**	-.909	38	.369	-.909	38	.369	.217	38	.829
**A4**	1.178	41	.245	1.178	41	.245	-1.867	41	.069
**Cholesterol**	.834	31	.410	.834	31	.410	-1.809	31	.080
**HDL**	.005	30	.996	.005	30	.996	-.151	30	.881
**Non-HDL cholesterol**	.971	30	.339	.971	30	.339	-2.032	30	.051
**LDL**	1.241	29	.224	1.241	29	.224	-2.275	29	.030
**Triglycerides**	.172	31	.864	.172	31	.864	-.362	31	.720
**Fasting Insulin**	-.734	39	.467	-.734	39	.467	.646	39	.522
**HOMA-IR**	-.985	39	.331	-.985	39	.331	.827	39	.413
**QUICKI**	.477	39	.636	.477	39	.636	-.524	39	.603
**log fasting insulin**	-.487	39	.629	-.487	39	.629	.551	39	.584
**Ovarian volume**	.009	32	.992	.009	32	.992	-.610	32	.546
**Follicle number**	1.199	31	.240	1.199	31	.240	-1.765	31	.087
**Menstruations per Year**	.430	39	.669	.430	39	.669	-.164	39	.870
**G/I**	.454	39	.652	.454	39	.652	-.688	39	.495
**IGF1**	-2.162	37	.037	-2.162	37	.037	2.334	37	.025

Ph C

	**APO A**			**APO B**				**LpA**	
**Variables**	t test	df	p-value	t test	df	p-value	t test	df	p-value
**AGE**	-.613	110	.541	-.566	110	.572	-.180	110	.857
**BMI**	-.002	106	.998	-.144	106	.885	.095	106	.924
**DHEAS**	2.619	108	.010	2.314	108	.023	-1.633	108	.105
**SHBG**	-.180	108	.857	.001	108	.999	-.774	108	.440
**FSH**	2.128	105	.036	2.230	105	.028	-2.009	105	.047
**LH**	-.493	105	.623	-.310	105	.757	.444	105	.658
**A4**	2.080	110	.040	1.797	110	.075	-3.195	110	.002
**Cholesterol**	-1.786	103	.077	-1.608	103	.111	.687	103	.493
**HDL**	-.311	100	.756	-.123	100	.902	.628	100	.531
**Non-HDL cholesterol**	-1.961	100	.053	-1.849	100	.067	.692	100	.490

**LDL**	-1.707	97	.091	-1.590	97	.115	.160	97	.873
**Triglycerides**	-1.198	99	.234	-1.156	99	.250	1.412	99	.161
**Fasting Insulin**	-.041	108	.967	-.225	108	.822	.598	108	.551
**HOMA-IR**	.151	108	.880	-.009	108	.992	.390	108	.697
**QUICKI**	.847	108	.398	1.054	108	.294	-1.516	108	.132
**log fasting insulin**	-.600	108	.550	-.832	108	.407	1.366	108	.175
**Ovarian volume**	-1.901	108	.060	-1.901	108	.060	.929	108	.355
**Follicle number**	1.497	108	.137	1.497	108	.137	-1.112	108	.269
**Menstruations per Year**	-.777	107	.439	-.570	107	.569	.954	107	.342
**G/I**	1.215	108	.227	1.379	108	.171	-1.872	108	.064
**IGF1**	-1.546	95	.125	-1.546	95	.125	2.286	95	.024

**Controls**									
	**APO A**			**APO B**				**LpA**	
**Variables**	t test	df	p-value	t test	df	p-value	t test	df	p-value
**AGE**	.523	48	.603	.590	48	.558	-.836	48	.407
**BMI**	.970	47	.337	1.188	47	.241	-2.622	47	.012
**DHEAS**	-.834	47	.408	-.575	47	.568	1.192	47	.239
**SHBG**	-.125	47	.901	-.017	47	.986	-1.021	47	.312
**FSH**	1.286	47	.205	1.097	47	.278	-2.152	47	.037
**LH**	.324	47	.747	.112	47	.911	-1.143	47	.259
**A4**	1.167	47	.249	1.302	47	.199	-1.866	47	.068
**Cholesterol**	-.067	42	.947	-.350	42	.728	.439	42	.662
**HDL**	-.827	40	.413	-1.442	40	.157	.709	40	.482
**Non-HDL cholesterol**	-.136	40	.892	-.208	40	.836	.516	40	.609
**LDL**	.459	39	.649	.395	39	.695	-.014	39	.989
**Triglycerides**	-1.296	40	.202	-1.416	40	.164	1.416	40	.164
**Fasting Insulin**	-.092	45	.927	-.534	45	.596	-1.340	45	.187
**HOMA-IR**	-.321	45	.750	-.732	45	.468	-1.275	45	.209
**QUICKI**	.099	46	.921	.379	46	.706	.621	46	.537
**log fasting insulin**	-.642	43	.524	-1.028	43	.310	-.740	43	.463
**Ovarian volume**	.927	40	.359	.927	40	.359	-.675	40	.503

**Follicle number**	.899	40	.374	.899	40	.374	-.110	40	.912
**Menstruations per Year**	2.410	48	.020	2.384	48	.021	-1.412	48	.164
**G/I**	-.388	45	.699	-.148	45.0	.883	.931	45	.356
**IGF1**	-2.306	41	.026	-2.096	41.0	.042	1.442	41	.157

Supplementary Table S2. Spearman Correlation and Partial Spearman correlation results (BMI and AGE as covariates).

		PhA			AGE and BMI corrected		PhB			AGE and BMI corrected		PhC			AGE and BMI corrected		CNT			AGE and BMI corrected	
Menstruations per year		N	Rho	p-value	Rho	p-value	N	Rho	p-value	Rho	p-value	N	Rho	p-value	Rho	p-value	N	Rho	p-value	Rho	p-value
	IGF1	120	.153	.095	.006	.949	37	.260	.120	.166	.340	95	-.135	.194	.009	.936	43	-.246	.112	-.225	.162
	Cholesterol	122	-.147	.106	-.088	.340	31	.243	.188	.314	.097	102	-.002	.986	-.034	.744	44	.051	.742	-.063	.696
	HDL	118	-.001	.988	-.016	.865	30	.457	.011	.443	.018	99	-.018	.863	.082	.431	42	-.023	.883	-.091	.581
	Non-HDL cholesterol	118	-.145	.117	-.095	.315	30	.188	.319	.291	.133	99	.020	.846	-.043	.681	42	.122	.442	.033	.842
	LDL	117	-.103	.268	-.059	.531	30	.136	.473	.209	.285	96	.006	.956	-.053	.618	41	.128	.425	.044	.792
	ApoA	92	-.058	.582	-.052	.630	18	.485	.042	.389	.137	63	-.023	.860	.065	.624	23	.250	.251	.135	.570
	ApoB	92	-.187	.074	-.132	.217	18	-.386	.113	-.243	.365	62	.092	.477	.025	.855	22	-.376	.084	-.509	.026
	ApoB / ApoA	92	-.172	.101	-.124	.247	18	-.487	.040	-.332	.209	62	.074	.569	-.021	.878	22	-.394	.069	-.455	.050
	Lp(a)	81	-.093	.408	-.097	.397	14	.322	.262	.403	.193	59	-.059	.655	-.079	.572	18	-.300	.227	-.186	.506
	Triglycerides	121	-.131	.153	-.091	.325	31	.211	.253	.304	.108	98	-.002	.984	-.034	.748	42	-.064	.688	-.140	.396
	Fasting Insulin	125	-.200	.026	-.188	.038	39	.138	.402	.217	.197	107	-.099	.312	-.069	.490	47	-.198	.183	-.229	.135
	HOMA-IR	117	-.144	.122	-.115	.225	39	.139	.400	.226	.178	107	-.115	.237	-.086	.395	47	-.211	.154	-.244	.110
	G/I	124	.221	.014	.225	.013	39	-.107	.518	-.161	.341	107	.077	.431	.032	.748	47	.185	.213	.222	.147
	Log Fasting Insulin	124	-.191	.034	-.182	.046	39	.138	.402	.217	.197	107	-.099	.312	-.069	.490	45	-.281	.062	-.311	.045
	QUICKI	124	.168	.062	.146	.110	39	-.139	.400	-.226	.178	107	.115	.237	.086	.395	48	.208	.155	.249	.099
FT	IGF1	116	.073	.433	.133	.162	35	.092	.598	.029	.873	77	-.066	.567	.019	.874	42	.173	.273	.052	.753
	Cholesterol	120	.275	.002	.244	.008	29	-.181	.348	-.169	.400	85	.224	.040	.235	.036	43	-.169	.279	.022	.893
	HDL	117	-.188	.042	-.066	.488	28	-.300	.121	-.335	.094	83	-.090	.417	.165	.149	41	-.253	.111	-.139	.406
	Non-HDL cholesterol	117	.338	.000	.285	.002	28	-.136	.491	-.121	.557	83	.261	.017	.173	.130	41	-.118	.463	.021	.901
	LDL	116	.316	.001	.268	.004	27	-.193	.334	-.182	.384	80	.281	.011	.205	.078	40	-.128	.432	-.009	.956
	ApoA	90	-.098	.358	-.019	.859	16	.179	.507	.004	.990	51	.071	.618	.191	.199	22	-.518	.013	-.374	.114
	ApoB	90	.254	.016	.228	.034	16	-.284	.287	-.378	.182	50	.211	.140	.085	.573	21	-.312	.169	-.219	.383
	ApoB / ApoA	90	.284	.007	.225	.036	16	-.259	.332	-.290	.315	50	.152	.292	-.077	.613	21	-.136	.556	-.132	.602
	Lp(a)	79	-.045	.692	-.072	.539	12	-.709	.010	-.717	.020	49	.278	.053	.137	.374	17	.261	.312	.154	.598

	Triglycerides	119	.213	.020	.081	.388	29	.071	.713	.094	.640	81	.172	.125	.021	.856	40	.136	.403	.194	.250
	Fasting Insulin	123	.338	.000	.217	.018	37	.125	.461	.045	.796	88	.315	.003	.019	.866	46	-.051	.738	-.157	.315
	HOMA-IR	114	.282	.002	.169	.077	37	.134	.429	.062	.725	88	.331	.002	.049	.659	46	-.054	.723	-.161	.303
	G/I	122	-.332	.000	-.218	.017	37	-.126	.458	-.048	.785	88	-.301	.004	.000	1.000	46	.046	.761	.147	.346
	Log Fasting Insulin	122	.331	.000	.210	.022	37	.125	.461	.045	.796	88	.315	.003	.019	.866	44	-.015	.926	-.144	.370
	QUICKI	122	-.312	.000	-.182	.047	37	-.134	.429	-.062	.725	88	-.331	.002	-.049	.659	46	.054	.723	.161	.303
A4	IGF1	121	-.037	.685	-.043	.648	39	-.279	.085	-.324	.051	97	-.113	.269	-.134	.201	42	-.189	.231	-.133	.420
	Cholesterol	126	-.115	.200	-.122	.178	33	.164	.362	.138	.460	105	.098	.320	.081	.425	43	-.118	.453	-.175	.280
	HDL	122	.043	.642	-.041	.657	32	.043	.814	.061	.749	102	.081	.416	.149	.146	41	-.177	.267	-.180	.280
	Non-HDL cholesterol	122	-.097	.288	-.072	.437	32	.168	.357	.142	.455	102	-.010	.921	-.089	.387	41	-.086	.591	-.108	.519
	LDL	121	-.108	.238	-.092	.322	31	.182	.327	.148	.443	99	.006	.950	-.062	.550	40	-.071	.662	-.074	.663
	ApoA	95	.046	.658	.011	.918	19	.412	.079	.308	.229	65	.017	.893	.048	.714	22	.017	.940	-.137	.577
	ApoB	95	-.043	.677	.040	.702	19	.031	.901	-.074	.777	64	.047	.712	-.059	.657	21	-.061	.792	.190	.451
	ApoB / ApoA	95	-.028	.790	.069	.514	19	-.139	.571	-.222	.392	64	.025	.845	-.088	.505	21	-.039	.867	.287	.248
	Lp(a)	84	.132	.232	.126	.264	15	.013	.965	.150	.625	61	-.073	.576	-.223	.098	17	.157	.546	.260	.370
	Triglycerides	125	-.068	.450	-.007	.939	33	-.065	.721	-.104	.579	101	.086	.395	.038	.716	41	-.103	.524	-.073	.661
	Fasting Insulin	129	-.038	.665	.074	.413	41	-.337	.031	-.337	.036	110	.069	.475	-.038	.700	46	-.073	.629	-.077	.622
	HOMA-IR	120	-.098	.287	.008	.933	41	-.360	.021	-.363	.023	110	.065	.502	-.045	.653	46	-.069	.647	-.068	.663
	G/I	128	-.016	.858	-.135	.133	41	.286	.070	.271	.095	110	-.074	.443	.024	.809	46	.052	.731	.049	.753
	Log Fasting Insulin	128	-.039	.666	.074	.413	41	-.337	.031	-.337	.036	110	.069	.475	-.038	.700	44	-.073	.636	-.070	.665
	QUICKI	128	.068	.443	-.034	.711	41	.360	.021	.363	.023	110	-.065	.502	.045	.653	47	.105	.484	.120	.439
DHEAS	IGF1	120	.117	.203	-.010	.913	39	.211	.197	.140	.409	96	.000	.999	.028	.794	42	.259	.098	.102	.538
	Cholesterol	125	-.106	.240	-.047	.610	33	-.337	.055	-.314	.086	103	-.181	.067	-.179	.077	43	.039	.806	.182	.262
	HDL	121	-.090	.329	-.129	.166	32	-.046	.802	-.022	.908	100	-.061	.545	-.052	.615	41	-.074	.646	-.042	.802
	Non-HDL cholesterol	121	-.089	.332	-.019	.835	32	-.367	.039	-.370	.044	100	-.144	.154	-.163	.115	41	.116	.471	.233	.159
	LDL	120	-.058	.533	-.006	.947	31	-.378	.036	-.380	.042	97	-.156	.128	-.159	.129	40	.032	.845	.115	.499
	ApoA	94	-.073	.481	-.053	.617	19	-.039	.872	.098	.709	65	-.196	.117	-.191	.140	22	-.272	.220	-.342	.151
	ApoB	94	-.111	.285	.032	.760	19	-.584	.009	-.562	.019	64	-.097	.447	-.063	.632	21	.091	.695	.205	.415
	ApoB / ApoA	94	-.060	.563	.061	.566	19	-.486	.035	-.469	.057	64	.021	.868	.059	.652	21	.135	.559	.262	.293
	Lp(a)	83	.230	.036	.224	.046	15	-.454	.089	-.449	.124	61	.183	.157	.148	.276	18	.305	.219	.322	.242

	Triglycerides	124	-.132	.143	-.057	.533	33	-.123	.494	-.136	.466	99	-.088	.386	-.097	.351	41	.171	.287	.242	.143
	Fasting Insulin	128	-.124	.165	-.100	.266	41	.012	.941	-.061	.710	108	.233	.015	.188	.058	46	.067	.658	.091	.562
	HOMA-IR	120	-.145	.114	-.113	.227	41	-.012	.943	-.081	.625	108	.218	.023	.170	.087	46	.066	.665	.084	.594
	G/I	127	.096	.282	.077	.393	41	.013	.938	.097	.555	108	-.251	.009	-.213	.031	46	-.054	.723	-.075	.635
	Log Fasting Insulin	127	-.115	.198	-.094	.299	41	.012	.941	-.061	.710	108	.233	.015	.188	.058	44	.158	.305	.185	.247
	QUICKI	127	.129	.150	.107	.237	41	.012	.943	.081	.625	108	-.218	.023	-.170	.087	47	-.075	.614	-.110	.477
SHBG	IGF1	117	.065	.487	.058	.542	37	-.128	.450	-.094	.590	97	-.114	.264	-.084	.424	42	-.081	.611	.041	.805
	Cholesterol	122	-.010	.914	.057	.536	30	.447	.013	.434	.021	103	.033	.740	.039	.699	43	.110	.481	.049	.764
	HDL	118	.573	.000	.425	.000	29	.328	.083	.272	.170	100	.263	.008	.129	.214	41	.391	.012	.419	.009
	Non-HDL cholesterol	118	-.211	.022	-.115	.220	29	.427	.021	.461	.016	100	-.068	.502	-.013	.899	41	-.087	.588	-.125	.456
	LDL	117	-.125	.178	-.040	.675	28	.351	.067	.368	.065	97	.028	.788	.072	.496	40	-.148	.362	-.160	.343
	ApoA	91	.330	.001	.162	.131	18	-.080	.754	-.195	.469	65	.075	.551	.057	.661	23	.370	.082	.303	.194
	ApoB	91	-.213	.043	-.170	.113	18	.303	.222	.384	.142	64	.035	.784	.047	.720	22	-.102	.650	-.049	.844
	ApoB / ApoA	91	-.325	.002	-.210	.050	18	.189	.453	.252	.346	64	.001	.995	.022	.869	22	-.232	.299	-.143	.560
	Lp(a)	80	-.004	.972	-.004	.976	14	.141	.631	.160	.620	61	-.064	.623	.002	.989	18	-.315	.203	-.282	.308
	Triglycerides	121	-.338	.000	-.150	.104	30	-.100	.600	-.086	.662	99	-.256	.011	-.166	.111	41	-.045	.782	-.043	.799
	Fasting Insulin	125	-.332	.000	-.031	.734	38	-.377	.020	-.110	.523	108	-.413	.000	-.173	.082	46	-.227	.129	-.223	.150
	HOMA-IR	117	-.357	.000	-.076	.421	38	-.377	.020	-.121	.483	108	-.408	.000	-.174	.080	46	-.232	.121	-.225	.147
	G/I	124	.274	.002	-.037	.690	38	.330	.043	.054	.754	108	.401	.000	.157	.115	46	.209	.164	.198	.204
	Log Fasting Insulin	124	-.330	.000	-.028	.757	38	-.377	.020	-.110	.523	108	-.413	.000	-.173	.082	44	-.238	.121	-.225	.158
	QUICKI	124	.346	.000	.058	.525	38	.377	.020	.121	.483	108	.408	.000	.174	.080	47	.265	.072	.277	.069
FSH	IGF1	119	-.284	.002	-.298	.001	36	-.032	.855	-.017	.923	92	-.207	.048	-.192	.074	43	-.043	.785	-.016	.922
	Cholesterol	123	-.058	.526	-.086	.351	31	-.026	.889	-.060	.757	100	.064	.524	.030	.774	44	.049	.754	.024	.881
	HDL	119	.128	.165	.115	.219	30	-.459	.011	-.434	.021	98	.008	.935	.008	.936	42	.030	.853	.061	.712
	Non-HDL cholesterol	119	-.072	.434	-.102	.278	30	.093	.625	.041	.836	98	.034	.743	-.004	.970	42	.056	.724	.039	.813
	LDL	118	-.002	.987	-.013	.894	29	.122	.527	.085	.674	95	.091	.381	.064	.546	41	.090	.577	.072	.667
	ApoA	92	.188	.073	.171	.109	19	-.114	.642	-.125	.634	63	-.006	.961	-.028	.833	23	-.090	.682	.143	.548
	ApoB	92	-.035	.740	-.054	.616	19	-.065	.791	-.136	.602	62	.039	.765	-.030	.822	22	.115	.609	.140	.567
	ApoB / ApoA	92	-.103	.329	-.113	.294	19	.020	.935	-.029	.911	62	.059	.650	.015	.908	22	.193	.390	.135	.581
	Lp(a)	82	.026	.815	.038	.740	15	.245	.379	.192	.529	59	.202	.125	.215	.115	18	-.022	.932	-.084	.767

	Triglycerides	122	-.151	.097	-.191	.037	31	.147	.429	.069	.721	96	-.054	.600	-.071	.504	42	-.139	.380	-.182	.269
	Fasting Insulin	126	-.076	.400	-.056	.539	38	-.023	.889	-.251	.139	105	-.034	.733	.005	.957	46	.084	.580	-.010	.950
	HOMA-IR	117	-.030	.746	-.031	.744	38	.000	.998	-.216	.205	105	-.015	.880	.022	.830	46	.087	.564	-.001	.993
	G/I	125	.059	.510	.025	.784	38	.035	.835	.248	.145	105	.052	.600	.015	.881	46	-.079	.602	.019	.902
	Log Fasting Insulin	125	-.082	.362	-.061	.507	38	-.023	.889	-.251	.139	105	-.034	.733	.005	.957	44	.087	.574	-.018	.912
	QUICKI	125	.091	.314	.078	.396	38	.000	.998	.216	.205	105	.015	.880	-.022	.830	47	-.049	.746	.037	.813
LH	IGF1	119	.003	.970	-.008	.934	36	-.046	.788	-.089	.615	92	.011	.914	.035	.749	43	-.090	.567	-.065	.688
	Cholesterol	123	-.152	.093	-.123	.180	31	.338	.063	.353	.060	100	-.027	.791	-.032	.757	44	.155	.315	.095	.556
	HDL	119	.224	.014	.109	.246	30	-.045	.812	-.048	.808	98	.012	.908	-.088	.399	42	.190	.229	.233	.153
	Non-HDL cholesterol	119	-.243	.008	-.178	.056	30	.313	.093	.337	.080	98	-.063	.538	-.036	.727	42	.230	.142	.142	.387
	LDL	118	-.192	.037	-.148	.115	29	.208	.278	.216	.280	95	-.061	.555	-.053	.621	41	.192	.230	.099	.555
	ApoA	92	.104	.326	.013	.904	19	-.148	.544	-.103	.693	63	-.166	.194	-.252	.052	23	.133	.545	.361	.118
	ApoB	92	-.083	.433	-.037	.733	19	.265	.273	.211	.416	62	-.081	.530	-.044	.742	22	.323	.142	.130	.596
	ApoB / ApoA	92	-.079	.454	.012	.912	19	.314	.190	.257	.320	62	-.012	.928	.070	.599	22	.341	.120	.054	.828
	Lp(a)	82	-.047	.674	-.047	.683	15	-.072	.800	-.113	.714	59	.160	.226	.186	.173	18	.307	.216	.271	.328
	Triglycerides	122	-.242	.007	-.138	.135	31	.291	.112	.318	.093	96	.067	.515	.133	.206	42	.086	.590	-.047	.778
	Fasting Insulin	126	-.212	.017	-.093	.304	38	-.092	.583	-.142	.408	105	-.217	.026	-.139	.167	46	.009	.952	-.134	.393
	HOMA-IR	117	-.257	.005	-.158	.093	38	-.096	.565	-.147	.391	105	-.206	.035	-.132	.190	46	-.031	.839	-.174	.265
	G/I	125	.161	.072	.030	.740	38	.033	.843	.057	.742	105	.223	.022	.148	.143	46	-.037	.809	.118	.452
	Log Fasting Insulin	125	-.220	.014	-.100	.273	38	-.092	.583	-.142	.408	105	-.217	.026	-.139	.167	44	.047	.760	-.092	.568
	QUICKI	125	.252	.005	.144	.113	38	.096	.565	.147	.391	105	.206	.035	.132	.190	47	.087	.559	.232	.129
Ovarian volume	IGF1	118	.064	.494	.076	.417	33	.147	.413	.045	.810	96	-.079	.444	-.046	.661	37	-.068	.689	-.214	.225
	Cholesterol	123	-.126	.164	-.126	.169	27	.102	.612	.132	.530	103	.143	.149	.059	.561	37	.175	.299	.279	.109
	HDL	119	-.249	.006	-.250	.007	26	-.038	.854	-.066	.759	100	-.119	.238	-.034	.742	35	.234	.175	.165	.366
	Non-HDL cholesterol	119	-.036	.699	-.034	.715	26	.091	.658	.136	.525	100	.192	.056	.087	.400	35	.160	.360	.305	.090
	LDL	118	-.097	.296	-.098	.299	25	.063	.763	.129	.558	97	.227	.025	.139	.184	35	.188	.281	.301	.095
	ApoA	92	-.228	.028	-.200	.061	15	.050	.860	-.372	.211	63	-.003	.981	.047	.720	18	.299	.227	.052	.854
	ApoB	92	.066	.534	.075	.487	15	-.284	.304	-.094	.759	63	.161	.206	-.046	.730	18	.015	.951	.270	.331
	ApoB / ApoA	92	.205	.050	.211	.047	15	-.136	.630	.160	.601	63	.146	.255	-.047	.720	18	-.100	.693	.230	.410
	Lp(a)	82	.090	.421	.082	.472	12	-.452	.140	-.620	.056	60	-.078	.554	-.144	.288	15	.286	.301	.477	.117

	Triglycerides	122	.124	.173	.127	.168	27	.084	.677	.100	.634	99	-.034	.735	-.152	.142	35	-.075	.670	.063	.733
	Fasting Insulin	126	.111	.215	.090	.323	33	-.002	.993	.151	.416	108	.030	.758	.025	.801	39	.228	.162	.329	.050
	HOMA-IR	117	-.022	.818	-.034	.722	33	.010	.956	.160	.390	108	.026	.792	.018	.854	39	.209	.202	.298	.078
	G/I	125	-.122	.176	-.098	.283	33	-.014	.939	-.163	.382	108	-.024	.805	-.018	.856	39	-.242	.137	-.343	.041
	Log Fasting Insulin	125	.115	.200	.093	.306	33	-.002	.993	.151	.416	108	.030	.758	.025	.801	37	.171	.313	.274	.117
	QUICKI	125	-.091	.314	-.061	.501	33	-.010	.956	-.160	.390	108	-.026	.792	-.018	.854	40	-.142	.382	-.194	.251
Follicle number	IGF1	116	-.221	.017	-.081	.394	32	-.100	.585	-.195	.301	96	-.073	.482	-.008	.938	37	-.154	.361	-.069	.697
	Cholesterol	121	.027	.767	-.072	.433	26	-.013	.948	-.041	.851	103	.046	.644	.027	.790	37	-.005	.975	-.109	.539
	HDL	117	-.127	.171	-.088	.349	25	-.001	.995	-.145	.510	100	.100	.320	.105	.308	35	-.049	.780	-.173	.344
	Non-HDL cholesterol	117	.073	.435	-.036	.700	25	-.010	.961	.011	.961	100	.006	.952	-.030	.769	35	.043	.805	-.009	.962
	LDL	116	.040	.672	-.047	.620	24	.016	.941	.055	.809	97	.020	.847	-.020	.852	35	.076	.663	.027	.885
	ApoA	90	-.123	.249	-.109	.311	15	.122	.665	-.174	.571	63	-.020	.877	.020	.881	18	.155	.540	-.281	.311
	ApoB	90	.271	.010	.176	.100	15	-.022	.938	.186	.544	63	.097	.449	.026	.846	18	-.077	.762	-.026	.926
	ApoB / ApoA	90	.305	.003	.215	.044	15	-.104	.713	.116	.707	63	.082	.523	.004	.979	18	-.096	.705	.033	.906
	Lp(a)	80	.102	.366	.103	.369	12	-.294	.354	-.368	.296	60	.223	.086	.123	.367	15	.047	.867	.192	.550
	Triglycerides	120	.143	.120	.028	.763	26	-.187	.360	-.140	.514	99	-.054	.595	-.070	.498	35	.018	.920	.022	.904
	Fasting Insulin	124	.057	.529	-.070	.442	32	-.520	.002	-.377	.040	108	-.020	.837	-.025	.799	39	.169	.305	.213	.213
	HOMA-IR	115	.052	.581	-.110	.246	32	-.509	.003	-.364	.048	108	-.028	.775	-.039	.698	39	.177	.280	.221	.196
	G/I	123	-.045	.622	.060	.517	32	.465	.007	.306	.100	108	-.004	.966	-.009	.930	39	-.182	.267	-.223	.192
	Log Fasting Insulin	123	.058	.526	-.070	.446	32	-.520	.002	-.377	.040	108	-.020	.837	-.025	.799	37	.073	.667	.121	.497
	QUICKI	123	-.059	.517	.077	.403	32	.509	.003	.364	.048	108	.028	.775	.039	.698	40	-.164	.313	-.184	.275

Supplementary Table S3. False Discovery Rate-corrected values and 95% Confidence intervals.

PhA	Correlation	N	Rho	uncorrected p-value	95% Confidence Interval:	Benjamini-Hochberg Adjusted P value
	FT - Cholesterol	120	.275427	.002329	[.0958 , .4377]	.04696
	FT - Non-HDL cholesterol	117	.337739	.000196	[.161 , .4935]	.007056
	FT - LDL	116	.315511	.000561	[.1359 , .475]	.01782
	FT - Fasting Insulin	123	.338497	.000128	[.1665 , .4905]	.007056
	FT - HOMA-IR	114	.282234	.002348	[.0982 , .4476]	.04696
	FT - G/I	122	-.332078	.000186	[-.4856 , -.1587]	.007056
	FT - Log Fasting Insulin	122	.331255	.000194	[.1578 , .4848]	.007056
	FT - QUICKI	122	-.312137	.000465	[-.4684 , -.137]	.015694
	SHBG - HDL	118	.572893	.00000	[.433 , .6858]	.0000
	SHBG - ApoA	91	.329732	.001414	[.1266 , .5063]	.04242
	SHBG - ApoB / ApoA	91	-.324531	.001698	[-.5019 , -.1209]	.043663
	SHBG - Triglycerides	121	-.338304	.000147	[-.4915 , -.1648]	.007056
	SHBG - Fasting Insulin	125	-.33226	.000153	[-.484 , -.1612]	.007056
	SHBG - HOMA-IR	117	-.356636	.000079	[-.5095 , -.1819]	.005603
	SHBG - G/I	124	.274195	.002058	[.0976 , .4341]	.04696
	SHBG - Log Fasting Insulin	124	-.329531	.000186	[-.4822 , -.1575]	.007056
	SHBG - QUICKI	124	.34596	.000083	[.1755 , .4963]	.005603
	FSH - IGF1	119	-.283515	.001781	[-.4454 , -.1037]	.043715
**PhB**						
	Follicle number - Fasting Insulin	32	-.520485	.002259	[-.7406 , -.1994]	.04696
	Follicle numbers - Log Fasting Insulin	32	-.520485	.002259	[-.7406 , -.1994]	.04696
**PhC**						
	FT - HOMA-IR	88	.331358	.001613	[.1247 , .5104]	.043551
	FT - QUICKI	88	-.331358	.001613	[-.5104 , -.1247]	.043551
	SHBG - Fasting Insulin	108	-.413282	.000009	[-.5626 , -.2379]	.001296
	SHBG - HOMA-IR	108	-.408253	.000012	[-.5584 , -.2322]	.001296
	SHBG - G/I	108	.40074	.000017	[.2237 , .5522]	.00153
	SHBG - Log Fasting Insulin	108	-.413282	.000009	[-.5626 , -.2379]	.001296

SHBG - QUICKI	108	.408253	.000012	[.2322 , .5584]	.001296

Note that the FDR correction was performed on 540 comparisons.

Figure S1. Histograms and detailed reports of the distributions of the follicle number for each group

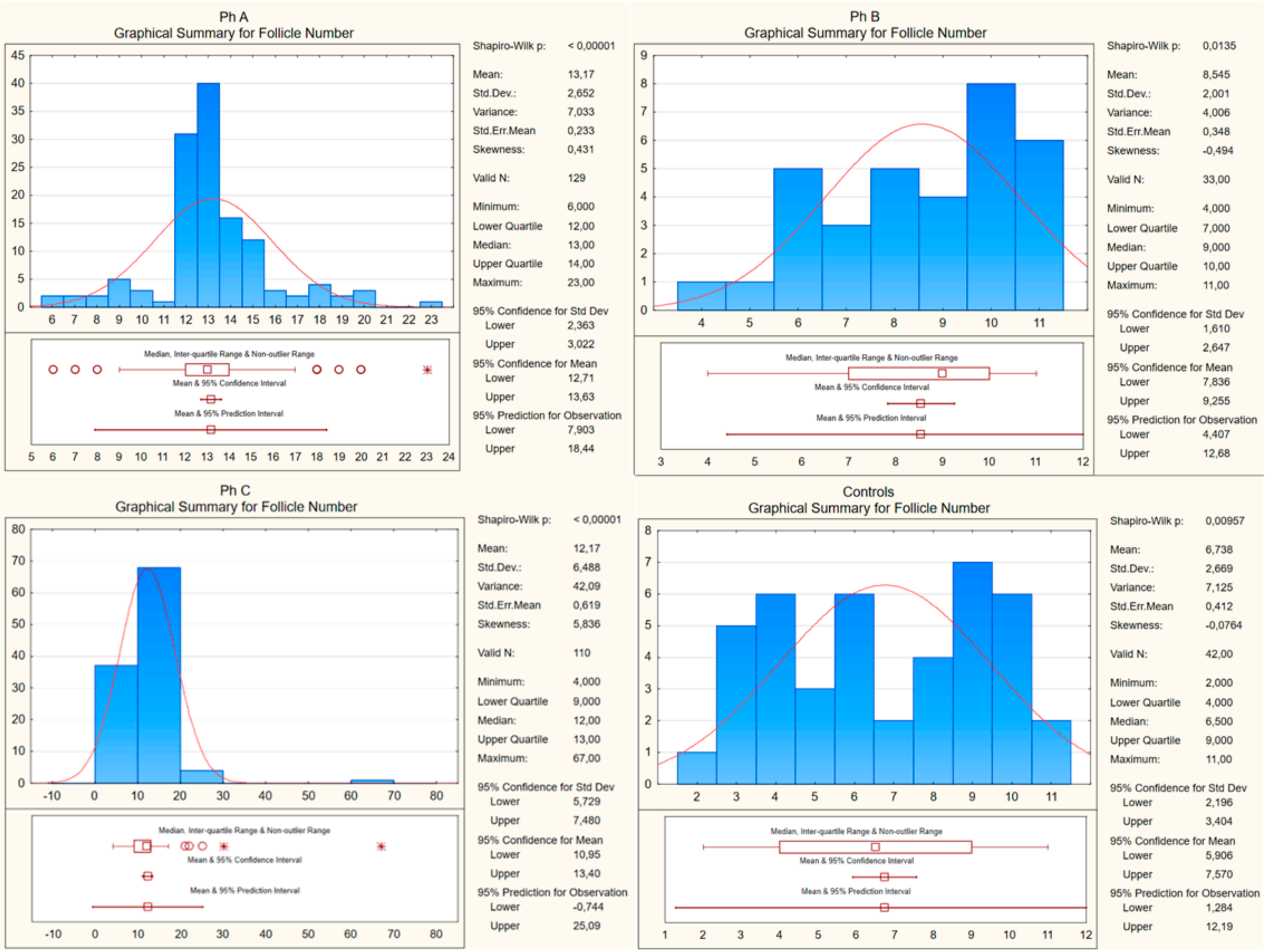

Note that the histograms were performed using Statistica (TIBCO vers. 14.0)
